# The moderating role of sex in the relationship between executive functions and academic procrastination in undergraduate students

**DOI:** 10.3389/fpsyg.2022.928425

**Published:** 2022-08-22

**Authors:** Lindsey W. Vilca

**Affiliations:** South American Center for Education and Research in Public Health, Universidad Norbert Wiener, Lima, Peru

**Keywords:** hot and cold executive functions, orbitofrontal cortex, medial prefrontal cortex, academic procrastination, undergraduate students, sex

## Abstract

The objective of the study was to determine if sex plays a moderating role in the relationship between executive functions and academic procrastination in 106 university students of both genders (28.3% male and 71.7% female) between the ages of 18 and 30 years (*M* = 19.7; SD = 2.7). The Academic Procrastination Scale and the Neuropsychological Battery of Executive Functions and Frontal Lobes (BANFE-2) were used to measure the variables. The results of the study showed that the degree of prediction of the tasks linked to the orbitomedial cortex (involves the orbitofrontal cortex [OFC] and the medial prefrontal cortex [mPFC]) on academic procrastination is significantly moderated by the sex of the university students (β_3_ = 0.53; *p* < 0.01). For men, the estimated effect of the tasks linked to the orbitomedial cortex on the degree of academic procrastination is −0.81. For women, the estimated effect of the tasks linked to the orbitomedial cortex on the degree of academic procrastination is −0.28. In addition, it was shown that sex does not play a moderating role in the relationship between the tasks linked to the dorsolateral prefrontal cortex (dlPFC) and academic procrastination (β_3_ = 0.12; *p* > 0.05). It was also determined that sex does not play a moderating role in the relationship between the tasks linked to the anterior prefrontal cortex (aPFC) and academic procrastination (β_3_ = 0.05; *p* > 0.05). It is concluded that only the executive functions associated with the orbitomedial cortex are moderated by the sex of the university students, where the impact of the tasks linked to the orbitomedial cortex on academic procrastination in men is significantly greater than in women.

## Introduction

In the university context, one of the most recurrent problems is academic procrastination where the student delays the development of their academic occupations voluntarily, usually doing it at the last minute (Steel et al., 2018). This construct can be defined as the voluntary delay of a planned, necessary and important activity, despite expecting possible negative consequences that outweigh the positive consequences of the delay (Steel, 2007; Klingsieck, 2013). In addition, this voluntary delay implies carrying out an alternative activity to the intended one and, therefore, is not synonymous with inactivity (H. C. Schouwenburg, 2004). Klingsieck (2013) raises seven characteristics of procrastination: (a) an intentional activity is delayed, (b) it is intended to start or end an activity, (c) The activity is necessary and of personal importance, (d) the delay is voluntary, (f) the delay is unnecessary, (g) the delay is made despite the negative consequences of the delay, and (h) the delay is accompanied by subjective discomfort or other negative consequences.

Several studies have shown that academic procrastination is present in all cultures, at all academic levels, and between genders (Steel, 2007; Klassen et al., 2008, 2010; Özer and Ferrari, 2011). In Turkey, a study on 784 university students showed that 52% frequently procrastinate (Özer et al., 2009). In China, a study of 1,184 university students reported that 74.1% procrastinate in at least one academic activity (Zhang et al., 2018). In Mexico, a study carried out on 521 psychology students from a public university showed that 57.9% have moderate academic procrastination (Chávez and Morales, 2017). In Peru, a study conducted on 517 psychology students from a private university showed that 14.1% have a high level of academic procrastination (Dominguez-Lara, 2017). The differences observed in the prevalence of academic procrastination could be explained by the sample size, the type of instrument used, and the methodology used to collect the data.

Regarding sexual differences in academic procrastination, there is an extensive discussion in the scientific literature due to the heterogeneity of the findings found in the different studies. Thus, several studies have found that there are significant differences between men and women in the level of academic procrastination (Özer et al., 2009; Steel and Ferrari, 2013; Mandap, 2016; Balkis and Duru, 2017). For example, a study conducted in Turkey on 441 university students found that men have higher levels of academic procrastination than women (Balkis and Duru, 2017). Another study in the Philippines with 200 university students showed that men procrastinate more than women (Mandap, 2016). Similarly, another study conducted in Turkey on 2,784 university students reported that men procrastinate more often than women (Özer et al., 2009). Another study on 16,413 English-speaking people showed that men are more likely to procrastinate (Steel and Ferrari, 2013).

However, other studies have not found significant differences between men and women (Sepehrian and Lotf, 2011; Zhou, 2020; Amoke et al., 2021). For example, a study conducted in China on 251 university students found no sex differences in academic procrastination (Zhou, 2020). Another study in Iran on 310 university students reported no significant differences between men and women in academic procrastination (Sepehrian and Lotf, 2011). Similarly, another study conducted in Nigeria on 804 people showed that gender does not significantly affect academic procrastination (Amoke et al., 2021). The heterogeneity of the results could be associated with methodological factors such as the size of the sample, the type of sampling used, and the measurement approach used. It could also be associated with cultural aspects.

That said, it was found that academic procrastination negatively affects the emotional well-being (Stead et al., 2010), life satisfaction (Özer and Saçkes, 2011) and even physical health (Sirois, 2015) of students. It is also related to the presence of anxious symptoms (Wang, 2021), high academic stress (Khalid et al., 2019), low self-esteem (Yang et al., 2021), and a greater presence of fraudulent academic behavior (Patrzek et al., 2015).

However, it is striking that despite the significant negative consequences of delaying their academic activities, most university students continue to procrastinate (Liu et al., 2020). This conduct could be explained by a failure to plan, regulate and control their behavior since they prioritize other secondary activities that imply more immediate gratification (Steel, 2007; Klassen et al., 2010; Park and Sperling, 2012). This behavior could also be explained by failing to self-regulate thoughts and emotions to maintain long-term behaviors such as studying for an exam or doing academic work (Steel and Ferrari, 2013). In this sense, emotional determinants such as impulsivity, emotional regulation, self-efficacy, motivation, and reward processing affect the level of academic procrastination (Wu et al., 2016; Wypych et al., 2018; Zhang et al., 2018; Mohammadi Bytamar et al., 2020). Cognitive determinants also affect this construct, such as planning, goal setting, metacognitive skills, and cognitive flexibility (Tan et al., 2008; Rabin et al., 2011; Ziegler and Opdenakker, 2018; Sutcliffe et al., 2019). Therefore, there are emotional and cognitive determinants that affect the level of academic procrastination. These determinants depend directly on the prefrontal areas of the brain associated with Executive Functions; specifically, these areas allow coordinating, selecting, and organizing various behavioral options to achieve goals that following procedures or rules can only obtain (Diamond, 2013). The review of the scientific literature shows that various components of Executive Functions such as self-control, planning, working memory, organization of materials, and task monitoring predict procrastination (Rabin et al., 2011). Also, impulsivity (Rebetez et al., 2018), self-efficacy, and self-control (Przepiórka et al., 2019) predict the level of procrastination. Likewise, evaluation-focused self-regulation is positively related to procrastination, and action-focused self-regulation is negatively related to procrastination (Choy and Cheung, 2018).

The Model of Hot and Cold Executive Functions could explain the emotional and cognitive determinants of academic procrastination since it distinguishes two domains of executive functions (Ward, 2020). (a) Hot functions are mostly related to emotional and motivational aspects (Salehinejad et al., 2021). It is also closely linked to reward processing, such as reward sensitivity and delay discounting (tendency to choose a smaller but more immediate reward over a larger but later reward) (Poland et al., 2016; Poon, 2018). Furthermore, it is linked to affective decision-making, social skills, theory of mind, empathy, and social cognition (Chan et al., 2008; De Luca and Leventer, 2008). Hot executive functions are associated with the medial and orbital regions of the prefrontal cortex (Salehinejad et al., 2021), which includes the orbitofrontal cortex (OFC) (McDonald, 2013; Baez and Ibanez, 2014) and the ventromedial prefrontal cortex (mPFC) (Zimmerman et al., 2016; Gazzaniga et al., 2019). Also, the medial region of the prefrontal cortex is crucial for emotional and motivational processing because it has connections with subcortical structures such as the limbic system, the amygdala, and the insular cortex (Sharpe and Shoenbaum, 2016; Matyi and Spielberg, 2021).

On the other hand, (b) cold functions are related to purely cognitive information processing, where their processes do not involve much emotional arousal and instead require a great deal of logical and critical analysis, where there is the conscious control of thoughts and actions (Chan et al., 2008; Rubia, 2011). In this domain, cognitive flexibility, inhibition, planning, working memory, verbal fluency, and problem-solving are involved (Poland et al., 2016; Nejati et al., 2018; Salehinejad et al., 2021). Attentional flexibility, concept formation, and the ability to monitor and adapt behavior according to changing social circumstances are also involved (Wood and Worthington, 2017). Cold executive functions are associated with the lateral region of the prefrontal cortex, which includes the dorsolateral prefrontal cortex (dlPFC) and the ventrolateral prefrontal cortex (Gazzaniga et al., 2019; Ward, 2020). A meta-analysis study carried out in 193 studies that used the magnetic resonance technique showed that the lateral region of the prefrontal cortex, the anterior cingulate cortex, and the parietal cortex were activated in the main domains of cold executive functions: working memory, inhibition, flexibility, and planning (Niendam et al., 2012). Another study also shows that these three regions are connected and are part of the fronto-cingulum-parietal network (FPN) that allows cognitive control, where the dlPFC plays a fundamental role (Salehinejad et al., 2021). It is important to note that both domains work together to perform adaptive functions, where emotional, social, and cognitive activities are involved (Zelazo and Carlson, 2012; Ruiz-Castañeda et al., 2020).

Then, understanding the fundamental role of executive functions in the initiation and maintenance of complex behaviors, it could be hypothesized that executive functions predict the degree of academic procrastination. However, the review of the literature also shows that the performance of executive functions in men and women is not the same (Silverman, 2003; Li et al., 2009; Nolen-Hoeksema, 2012; Weis et al., 2013; Weafer and de Wit, 2014; Gaillard et al., 2021b).

Several studies show that women have a greater capacity for delayed gratification (Weafer and de Wit, 2014) and greater behavioral self-regulation than men (Weis et al., 2013). In addition, women have a greater ability to use executive skills associated with controlling emotional reactions, cognitive reappraisal, and emotional coping (Nolen-Hoeksema, 2012). Women also use emotional regulation strategies to a greater extent and are more flexible in implementing these strategies (Goubet and Chrysikou, 2019). In contrast, men tend to avoid or repress emotional experiences (Barrett and Bliss-Moreau, 2009) and have greater problems with impulsivity (Riley et al., 2016). A meta-analysis study showed sex differences in the delay discount task (Gaillard et al., 2021a). Specifically, they found that women performed better than men, with a high effect size (Hedges’ *g* = 0.64). Women had a greater ability to discriminate and choose larger and later rewards than smaller and more immediate rewards. Similarly, another meta-analysis study conducted in 102 studies showed that women outperform men in delay capacity (Hedges’ *g* = 0.25–0.26) (Silverman, 2021).

However, in the scientific literature, there are also meta-analysis studies that show that there are no sex differences in tasks associated with executive functions, such as the study by Cross et al. (2011), where it was shown that there were no sex differences in the late discount. Similarly, another meta-analysis showed no sex differences in the ability to delay gratification (Silverman, 2003). Another systematic review study found little support for significant differences between men and women in executive function performance (Grissom and Reyes, 2019). The heterogeneity of these results could be associated with aspects such as the type of measurement used, cultural aspects, and specific characteristics of the sample.

On the other hand, sexual differences have also been studied using neuroimaging techniques. A study by Li et al. (2006) showed that men need more neural resources (greater activation of the bilateral medial frontal cortex, cingulate cortex, globus pallidus, thalamus, and parahippocampal gyrus) to have a similar performance to women in stop sign tests, which suggests greater impulsiveness in men. Another follow-up study by the same authors found that women have greater performance control and a greater effective response to error (Li et al., 2009). Also, several studies found sex differences in the middle, superior, and inferior frontal gyrus and OFC, which are involved in response inhibition capacity (Li et al., 2009; Gaillard et al., 2020).

On the other hand, neurological structures such as the mPFC and the amygdala, associated with emotional processing and decision making, follow different patterns of functional lateralization in men and women (Reber and Tranel, 2017). In women, decision-making and emotional processing are linked to the left side of the mPFC, while in men, it is linked to the right side of the mPFC (Reber and Tranel, 2017). In addition, women show a greater volume of mPFC and right OFC (Welborn et al., 2009).

A possible explanation for sex differences in the performance of executive functions can be partially explained by sex differences in the controllability of structural brain networks (Cornblath et al., 2019). A systematic review study of twenty-one neuroimaging studies showed sexual differences in the neural networks that underlie all executive control tasks (Gaillard et al., 2021b). This result suggests that men and women use different strategies depending on the task’s demands. Similarly, other studies have shown that the sex differences observed in executive functions could be partly explained by the experiences and cognitive strategies used by women and men (Satterthwaite et al., 2015; Wierenga et al., 2019).

For all the above, it can be affirmed that there is more evidence in the scientific literature in favor of differences between men and women in the performance of executive functions. Also, the functional and structural differences associated with executive functions could explain why men who procrastinate have higher levels of impulsivity (Strüber et al., 2008), lower levels of self-regulation (Higgins and Tewksbury, 2006), lower levels of self-motivation (Franklin et al., 2018) and greater problems in planning, monitoring and evaluating academic tasks (Limone et al., 2020). Unlike women who procrastinate, who have greater problems regulating cognitive and meta-cognitive processes (Limone et al., 2020).

**FIGURE 1:**
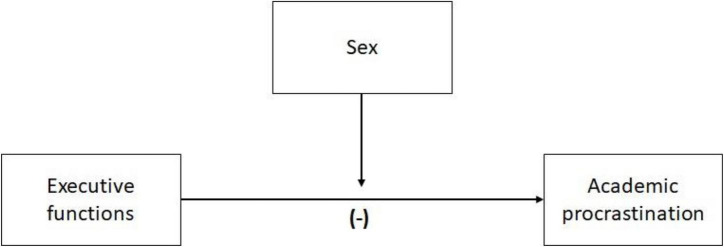
Hypothetical model: moderating role of sex.

Based on the above, it could be hypothesized that executive functions significantly predict the degree of academic procrastination and that gender plays a moderating role in the relationship between both variables (see Figure 1).

It is important to mention that most studies that assess the relationship between executive functions and academic procrastination use self-report scales to assess executive functions (Rabin et al., 2011; Sabri et al., 2016; Gutiérrez-García et al., 2020), which constitutes an important limitation since they depend directly on the perception that those evaluated have of their capacities. Also, although most studies use samples of university students, they do not precisely measure academic procrastination since they use scales that measure procrastination in general. Responding to this need, this study proposes the following specific hypotheses: (1) the functions linked to the orbitomedial cortex significantly predict academic procrastination. (2) Sex plays a moderating role between functions linked to the orbitomedial cortex and academic procrastination. (3) Functions linked to the dlPFC significantly predict academic procrastination. (4) Sex plays a moderating role between functions linked to the dlPFC and academic procrastination. (5) Functions linked to the anterior prefrontal cortex (aPFC) significantly predict academic procrastination. (6) Sex plays a moderating role between functions linked to the aPFC and academic procrastination.

## Materials and methods

### Participants

In the present study, the sample consisted of 106 university students of both sexes (28.3% men and 71.7% women) between the ages of 18 and 30 years (*M* = 19.7; DS = 2.7) who were in the first and second year of Psychology at a private university in Lima, Peru. For data collection, a non-probabilistic convenience sampling was used, and the following inclusion criteria were used: (a) students who have signed the informed consent, (b) students over 18 years of age, and (c) students who are enrolled in the academic cycle of the university. The following exclusion criteria were also used: (a) Students who did not complete the two evaluation sessions, (b) Students who had some physical or sensory limitation that prevented them from answering the instruments on their own, and (c) Students who did not complete both tests. A *post hoc* procedure was performed to estimate statistical power, for which the following criteria were used: (a) effect size, (b) probability of error, (c) sample size, and (d) number of predictors. The statistical power was 0.98, considered adequate to estimate the regression models.

### Measures

#### Neuropsychological battery of executive functions and frontal lobes

The battery was developed by Flores Lázaro et al. (2008, 2012) to evaluate functions associated with the orbitomedial cortex (formed by the OFC and the mPFC), the dlPFC and the aPFC. In addition, the authors of the battery, following anatomical-functional criteria, selected a set of tests to measure these functions. For the OFC and the mPFC, the following tests were used: Stroop Effect (form A and B), Card Game, Mazes (traversing), and Card Classification (Maintenance Errors). The Stroop test measures inhibitory control capacity. In addition, several neuroimaging studies have shown that this test is associated with OFC and CPFM (Adleman et al., 2002; Jourdan Moser et al., 2009; Song and Hakoda, 2015; Cipolotti et al., 2016). The card game test is an adaptation of the Iowa Gambling test and assesses the ability to detect and avoid risky selections and to detect and maintain good selections. Several studies have found this test to be associated with OFC and mPFC (Bolla et al., 2004; Aram et al., 2019; Zha et al., 2022). On the other hand, the maze test assesses the ability to plan, respect limits, and follow the rules. The test primarily involves orbitofrontal and dorsolateral areas (Stevens et al., 2003; Thonnard et al., 2021). To evaluate the orbitofrontal areas, the traversing qualification criterion was used. The other qualifying criteria were used to measure CPFM.

For the dlPFC, the following tests were used: Self-Directed Pointing, Visuospatial Working Memory, Alphabetical Ordering of Words, Card Sorting (perseveration and timing), Mazes (planning and timing), Tower of Hanoi (three and four disks), Consecutive Addition and Subtraction (form A and B), Verbal Fluency and Semantic Classification. The Self-Directed Pointing Test assesses the ability to use visuospatial working memory to self-directed point to a series of figures. It mainly involves dorsolateral prefrontal areas, especially their ventral portions (Lamar and Resnick, 2004). The visuospatial working memory test assesses the ability to maintain the identity of objects located in a specific order and space. It is based on the Corsi cube test but introduces the variant proposed by Goldman-Rakic et al. (1996) and Petrides (2000) of pointing to figures that represent real objects. The test is associated with the dlPFC (Ross et al., 2007). The alphabetical order of words tests measures the mental ability to manipulate and order verbal information in working memory. Performance on this test is also associated with the dlPFC (Tsukiura et al., 2001; Tsujimoto et al., 2004). The Card Sorting Test is based on the Wisconsin Card Sorting Test and assesses a person’s mental flexibility. Performance on this test is directly related to dlPFC (Lie et al., 2006; Gläscher et al., 2019).

The maze test also allows the ability to systematically anticipate (plan) visuospatial behavior, which is associated with dlPFC (Kaller et al., 2011; Kronovsek et al., 2021). Specifically for this test, time and dead-end planning errors are considered. The Tower of Hanoi test assesses the ability to plan a series of actions that only together and in sequence lead to a specific goal (sequential planning). Performance on this test is associated with the dlPFC (Ruiz-Díaz et al., 2012; Niki et al., 2019). The addition and subtraction task evaluates the ability to perform simple calculation operations in reverse sequence both within and between tens. Performance on this test is associated with the dlPFC (Burbaud et al., 1999; Barahimi et al., 2021). Finally, the verbal fluency test measures the ability to efficiently select and produce as many verbs as possible within a limited time. Performance on this test is linked to the dlPFC (Akiyama et al., 2018; Panikratova et al., 2020).

For the aPFC, the following tests were used: Semantic classifications (number of abstract categories), Selection of proverbs, and Metamemory. The Semantic Classification test measures the ability to produce the greatest number of abstract categories (abstract attitude). The performance in this test mainly involves areas of the aPFC (Koenig et al., 2005; Matsumoto et al., 2021). The Proverbs Selection Test assesses the ability to understand, compare, and select figurative responses. Performance on this test is associated with aPFC (Thoma and Daum, 2006; Ferretti et al., 2007). Finally, the metamemory task measures the ability to develop a memory strategy (metacognitive control), as well as to make performance prediction judgments (metacognitive judgments) and adjustments between performance judgment and actual performance (metacognitive monitoring). Performance on this test is linked to the aPFC area (Kikyo et al., 2002; Chua et al., 2014).

The qualification process of the BANFE-2 battery was carried out in two stages: First, the scores of each one of the tests were obtained following the qualification norms given in the test manual. That is, a score was obtained for the criteria of each test. Only in some criteria was the original score coded in a range of 1 to 5 points depending on the age and schooling of the person evaluated. Second, the scores by cortical area (Orbitomedial, Dorsolateral, and aPFC) were obtained by adding the associated criteria for each area. These scores are the ones used for the regression models. It is important to mention that the entire qualification process was carried out following the instructions given in the test manual (Flores Lázaro et al., 2012). A detailed description of the associated areas, domains, tests, and their grading system can be seen in Table 1.

**TABLE 1:** Structure of the BANFE-2 neuropsychological battery.

Associated Area	Domain	Test	Description	Qualification criteria^a^	Time
Orbitomedial Cortex	Inhibitory control	Stroop Form A	The subject is asked to read what is written, except when the word is underlined, in which case they are asked to name the color in which it is printed and not what is written.	Stroop errors: when the underlined word is said instead of the color	5 min
				Time: time in seconds used to complete the test.	
				Correct answer: words read correctly. The maximum possible score is 84	
		Stroop Form B	The evaluator points to the columns of words printed in color and asks the subject to read what is written, but when the evaluator says the word “color,” the subject must name the color in which the words are printed not what is written.	Stroop errors: when the color in which the word is written is not mentioned in a column where it was instructed to mention the color.	5 min
				Time: time in seconds used to complete the test.	
				Correct answer: words read correctly. The maximum possible score is 84	
	Follow rules	Maze test	It is made up of five labyrinths that increase their level of difficulty. The subject is asked to solve the mazes in the shortest time possible, without touching the walls or going through them, and to try not to pick up the pencil once he has started. The number of times he touches the walls passes through them, and enters a dead end (planning error) is recorded. Likewise, the execution time is recorded.	Go through: number of times it goes through walls. It is considered that a wall has been crossed when the pencil line crosses any wall of the maze.	4 min
		Card Sorting	It consists of a base of four cards with four different geometric figures (circle, cross, star, and triangle), which have two properties: number and color. The subject is provided with a group of 64 cards with these same characteristics, which he has to accommodate under one of the four base cards presented on a sheet using a criterion that the subject has to generate (color, shape, or number). Any card has the same possibility of relating to the three criteria since no perceptual pattern guides decision-making.	Maintenance errors: When the correct sequence is not maintained, and it is decided to change the classification criteria after at least three consecutive hits.	10 min
	Risk-Taking processing	Card game	This test consists of choosing each card according to its criteria, taking into account the risks and benefits of the choice to achieve the greatest number of points possible. The stimuli of the cards are numbers that go from 1 to 5 and represent points. Cards 1, 2, and 3 have minor penalties and appear less frequently. The cards with more points (4 and 5) have more expensive and more frequent punishments. The points obtained are recorded, as well as the percentage of risk, which results from averaging the selections of cards 4 and 5.	Percentage of risk cards: it is obtained from the total number of cards that the person takes and the number of risk cards (4-point cards plus 5-point cards) taken. Total score: it is obtained by subtracting penalty points from earned points. The scores obtained may contain negative values (for example, −5), indicating that you have chosen a significant number of risk cards.	5 min
Dorsolateral Prefrontal Cortex (dlPFC)	Verbal fluency	Verbal fluency	The test considers the ability to generate verbs in a limited time.	Hits: total number of correctly mentioned verbs, not including intrusions or perseverations.	1 min
				Perseverations: mentioning the same verb two or more times	
	Mental flexibility	Card Sorting	It consists of a base of four cards with four different geometric figures (circle, cross, star, and triangle), which have two properties: number and color. The subject is provided with a group of 64 cards with these same characteristics, which he has to accommodate under one of the four base cards presented on a sheet using a criterion that the subject has to generate (color, shape, or number). Any card has the same possibility of relating to the three criteria since no perceptual pattern guides decision-making.	Hits: correspondence between the classification principle established by the test and the subject’s classification criteria. Perseverations: occur when the card placement immediately after an error corresponds to the same wrong criteria. Deferred perseverations: occur when the same wrong criterion chosen in any of the four previous attempts is used without considering the immediately preceding classification principle. Time: time in seconds used to complete the test.	10 min
	Visuospatial planning	Maze test	It is made up of five labyrinths that increase their level of difficulty. The subject is asked to solve the mazes in the shortest time possible, without touching the walls or going through them, and to try not to pick up the pencil once he has started. The number of times he touches the walls passes through them, and enters a dead end (planning error) is recorded. Likewise, the execution time is recorded. It also allows systematically assessing the ability to anticipate (plan) visuospatial behavior.	Dead-end planning: number of times the evaluated person enters a dead-end road. The choice of the wrong path does not need to lead to hitting a wall; the error is counted when the erroneous route takes more than half of the way. Time: the time is recorded since the indication to start solving the maze is given.	4 min
	Sequential planning	Tower of Hanoi 3 and 4 disks	It is made up of a wooden base with three stakes and three or four chips of different sizes. The task has three rules: - Only one of the checkers can be moved at a time. - A smaller checker cannot be under a larger checker. - Whenever a checker is taken, it must be deposited again before taking another. The subject has to move a pyramid-shaped configuration from one end of the base to the other by moving the tiles along with the pegs.	Movements: number of movements made until each task’s final goal. The minimum number of moves to correctly complete the three-disk problem is seven; for the task with four disks, it is 14 movements. Time: Time in seconds that it takes to complete the task. Both ratings are used separately for each tower.	4 min
	Reverse sequence	Consecutive subtraction A and B	In both cases, it is requested that from an indicated number (40 or 100), an amount be subtracted consecutively (three in three or seven in seven, respectively) until reaching the minimum number (two or one). Task A (40–3) applies from 8 years of age. Task B (100–7) only applies from 10 years of age.	Time: time in seconds elapsed from the time “begin” is said until the conclusion of the consecutive subtractions. Hits: the number of correct individual subtractions made by the person is considered. The maximum possible number of correct answers is 14 for the subtraction of 100–7 (task B) and 13 correct for the subtraction of 40–3 (task A). It is not recorded in the protocol if the person mentions 100 or 40 when starting to subtract.	5 min per task
		Consecutive sum	This task consists of developing a consecutive sum exceeding the tens limit. The following instruction is given: “we are going to do a sum. Starting from one, you have to add five by five; I will tell you when to stop.” The person is instructed to stop when signaled. It is stated that he cannot use his fingers.	Time: time in seconds from when the person is told to start until the end of the test. Hits: the number of correct individual sums is taken into account. The maximum possible number of hits is 20. It is not recorded in the protocol if the person mentions the one when starting to add	5 min
	Productivity	Semantic classification	Assesses the ability to analyze and group a series of animal figures into semantic categories in the largest possible number of categories. The subject is presented with a sheet with 30 animal figures and is asked to generate as many classifications as possible within 5 min.	Total Categories: total average number of items included in all categories. Total average of animals: the total of animals classified in some category is scored. Total score: one point is awarded if the category is Concrete (C), two are given if the category is Functional (F), and three if the category is Abstract (A). Points are awarded for each category generated, and scores are noted in the box on the left. The total score is the sum of the points given to each generated category. The maximum score is 36.	5 min
	Self-directed visual working memory	Self-directed pointing	The self-directed working memory test (WM) is made up of a sheet with figures of objects and animals. The goal is to point your finger at all the figures without omitting or repeating any. The subject has to develop an action strategy and, at the same time, maintain in his WM the figures that he has already pointed out so as not to repeat or omit any (persevere or omit in the indications).	Perseverations: figures indicated more than once. The figure is marked with the corresponding number and will be counted as a perseveration. Time: time in seconds used to finish pointing out the figures on the sheet. Hits: the total number of hits will be the number of figures indicated in a non-contiguous manner that has not been persevered. If the person points to two contiguous figures at first, the second will not be considered correct. From 12 indicated figures, whether they are correct or not, a marked figure that is contiguous to the previous figure can be counted as a hit.	5 min
	Verbal working memory-ordering	Alphabetical ordering of words	The test consists of three disyllabic word lists, the first containing words that begin with a vowel, the second with a consonant, and the last, with vowels and consonants. The task is to reproduce each list in alphabetical order. Assesses the ability to hold information in the WM and manipulate it mentally.	The following aspects are rated on each list: - Rehearsal number in which the list is played correctly. - Perseverations: perseverations are words that the person repeats more than once in an essay. - Intrusions: intrusions are words that the person mentions but are not on the list. - Order errors: Reproduce words whose initial vowel or consonant does not correspond to the alphabet sequence. These errors are scored on words provided and not omitted. A score is obtained for each list.	There is no time limit
	Visuospatial-sequential working memory	Visuospatial working memory	The task consists of four lists that increase the number of figures from four to seven elements. The order of the figures in each list is noted in the protocol. Two trials are provided for each word list. If the correct sequence is signaled on the first trial, it goes directly to the next level. The second trial applies only in case of failure to point to the figures on the first trial. The test is over if the person fails to signal the correct sequence on both trials.	Maximum sequence: corresponds to the maximum level indicated. The test is suspended due to two consecutive tests mismarked; the maximum sequence will correspond to the maximum level correctly marked. The maximum possible level is four. Perseverations: when a figure is pointed to more than once in a trial, either a correct figure or a substitution. Order errors: when a figure is indicated in the order that does not correspond to it according to the original sequence.	There is no time limit
Anterior Prefrontal Cortex (aPFC)	Metamemory	Metamemory	This test consists of memorizing a list of nine bisyllabic words during five trials and comparing it with the predicted performance of the evaluated person. The following instruction is given: “in the next task, I am going to read you a list of nine words; how many words do you think you can learn?” The number of words said by the person is recorded on the prediction line. When the person finishes saying the words that he managed to learn, the total number of words that he managed to memorize is mentioned to him, and he is told: “Now, I am going to read you the same words in the same order, how many words do you think you can learn?”. Continue in this way until you have completed all five trials, even if you have learned all the words before reaching the fifth trial.	Error: they are obtained by subtracting the predicted number of words and the number of words said in each trial. Two types of errors are scored separately: - Positive errors result from overestimating the number of words predicted by the subject. - Negative errors result from the underestimation of learning	There is no time limit
	Comprehension of figurative meaning	Selection of sayings	This test is made up of five sayings, for which three possible response options are presented.	Time: time in seconds to finish the test. Hits: the maximum possible score is five points. Every correct answer is worth one point.	5 min
	Abstract attitude	Semantic classification	Assesses the ability to analyze and group a series of animal figures into semantic categories in the largest possible number of categories. The subject is presented with a sheet with 30 animal figures and is asked to generate as many classifications as possible within 5 min.	The number of abstract categories: they define semantic-abstract properties of animals (mammals, domestic, marine, etc.).	5 min

^a^For some areas and domains, the same test is used, but different aspects of the qualification are considered.

#### Academic procrastination scale (APS)

The instrument was developed by Busko (1998) to measure the degree of academic procrastination in university students. For the study, the version adapted to Peru was used, where the two-dimensional model presented adequate fit indices (RMSEA = 0.079; CFI = 1.00; GFI = 0.97) and adequate reliability indices, both for the academic self-regulation dimension (ω = 0.83) and the postponement of activities (ω = 0.75) (Dominguez-Lara et al., 2014). The scale’s factor structure was confirmed in another study by the same author (Dominguez-Lara, 2016). Regarding the scale structure, the 12 items form two dimensions: academic self-regulation (2, 5, 6, 7, 10, 11, 12, 13, and 14) and postponement of activities (1, 8, and 9). In addition, the items have five response categories ranging from: “Never” (1) to “Always” (5), where a higher score on the scale shows greater academic procrastination. In the sample used, the global scale presents adequate internal consistency indices (α = 0.79; ω = 0.80).

### Procedure

The standards given in the Declaration of Helsinki for the study were followed (World Medical Association, 2013). Among these, the following principles were emphasized: (a) autonomy of the people to participate in the study, (b) respect toward the participants, (c) beneficence, and (d) justice to treat the participants with fairness and transparency. In addition, the study had the approval of the Institutional Research Ethics Committee (CIEI) of a private university in Lima (204085), and informed consent was also used for the participation of people in the study.

A non-probabilistic sample was used for data collection, and the instruments were applied individually in an evaluation room. For both tests, we had the help of three fifth-year psychology students, who received training for six sessions in the application of the test. A psychologist with a Master’s degree in Psychology and a specialty in Neuropsychology directed the training of the evaluators. During the evaluation process, the anonymity and confidentiality of the results were ensured, where the study’s objectives were explained to the university students, doubts related to the procedure were resolved, and they signed informed consent. In addition, the tests were applied in two sessions of approximately 35 min.

### Statistical analyses

To determine whether gender plays a moderating role between the relationship between executive functions and academic procrastination, a hierarchical regression analysis was used following the procedures described by Aiken et al. (1991). In addition, estimated marginal means (EMMs) of academic procrastination at different levels of executive functions by gender were calculated. Executive function effects were also tested separately for males and females with a simple slope analysis.

For the simple linear regression models, the following equation was used:


$$Y=\beta_{0}+\beta_{1}*X+\epsilon$$

Where β_*1*_ is the slope. In model 1, it is associated with the tasks linked to the orbitomedial cortex. In model 2, it is associated with the tasks linked to the dlPFC. In model 3, it is associated with the tasks linked to the aPFC.

For the moderation analyses, the following equation was used:


$$Y=\beta_{0}+\beta_{1}*X+\beta_{2}*Z+\beta_{3}*X*Z+\epsilon$$

$$Y=\beta_{0}+\beta_{1}*X+\epsilon formalestudents\left(Z=0\right)$$

$$Y=\beta_{0}+\beta_{2}+\left(\beta_{1}+\beta_{3}\right)*X+\epsilon forfemalestudents\left(Z=1\right)$$

Where β_*1*_ represents the estimated effect of the tasks linked to the orbitomedial cortex (model 1), the tasks linked to the dlPFC (model 2), and the tasks linked to the aPFC (model 3) on academic procrastination for the male group.

All statistical analyzes were performed using the “lm()” function for hierarchical regression and the “emmeans” package (Russell et al., 2021). The RStudio environment (RStudio Team, 2018) for R (R Core Team, 2019) was used in both cases.

## Results

### Descriptive analysis

Table 2 shows the descriptive analysis and the relationship between the study variables. In the total sample, it is evident that the tasks linked to the medial orbital cortex have a negative relationship with university students’ degree of academic procrastination (*r* = −0.59). However, the degree of academic procrastination does not show a relationship with the tasks linked to the dlPFC (*r* = 0.09) and the aPFC (*r* = −0.00).

**TABLE 2:** Descriptive analysis and correlation between variables.

Variables	*M*	*SD*	*Min*	*Max*	1	2	3	4
**Total sample**								
1. Orbitomedial Cortex	187.5	13.8	105	203	1	−0.05	−0.00	−0.59
2. Dorsolateral Prefrontal Cortex	195.0	32.9	135	459		1	0.12	0.09
3. Anterior Prefrontal Cortex	19.5	6.3	12	53			1	−0.00
4. Academic procrastination	27.7	7.2	12	46				1
**Male sample**								
1. Orbitomedial Cortex	189.0	6.1	176	199	1	0.25	0.06	−0.71
2. Dorsolateral Prefrontal Cortex	193.6	18.8	135	230		1	0.29	−0.22
3. Anterior Prefrontal Cortex	19.2	6.8	13	53			1	−0.03
4. Academic procrastination	29.1	6.9	20	46				1
**Female sample**								
1. Orbitomedial Cortex	186.9	15.9	105	203	1	−0.09	−0.01	−0.62
2. Dorsolateral Prefrontal Cortex	192.9	22.3	143	268		1	0.06	0.14
3. Anterior Prefrontal Cortex	19.5	6.1	12	51			1	0.01
4. Academic procrastination	27.2	7.3	12	43				1

Regarding the male sample, it is evident that the tasks linked to the orbitomedial cortex negatively correlates with the degree of academic procrastination (*r* = −0.71). It can also be seen that the tasks linked to the dlPFC has a negative and weak relationship with the degree of academic procrastination (*r* = −0.22). However, the degree of academic procrastination does not show a relationship with the tasks linked to the aPFC (*r* = −0.03). Regarding the sample of women, it can be seen that the tasks linked to the orbitomedial cortex has a negative relationship with the degree of academic procrastination (*r* = −0.62). It is also seen that the tasks linked to the dlPFC has a weak relationship with the degree of academic procrastination (*r* = 0.14). However, the degree of academic procrastination does not show a relationship with the tasks linked to the aPFC (*r* = 0.03). Therefore, it can be seen that the strength of the relationship between the orbitomedial cortex and academic procrastination varies in the groups of men and women.

### Hypothesis test of the explanatory model

Table 3 shows the results of the analysis of the interaction of the sex of the university students on the relationship between executive functions and academic procrastination.

**TABLE 3:** Model of the moderating effect of sex in the relationship of the variables.

	Academic procrastination					
	
	*β [IC95%]*	*t*	*p*	Δ*R*^2^	*p*	*f* ^2^
Model 1: stage 1				0.34	0.000*	0.52
Orbitomedial Cortex	−0.31 [−0.39–0.23]	−7.42	0.000*			
Model 1: Stage 2				0.41	0.000*	0.69
Orbitomedial Cortex	−0.81 [−1.15–0.47]	−4.75	0.000*			
Orbitomedial Cortex × Sex	0.53 [0.18–0.87]	2.99	0.000*			
Model 2: Stage 1				−0.01	0.558	0.11
Dorsolateral Prefrontal Cortex	0.02 [−0.05–0.09]	0.59	0.558			
Model 2: Stage 2				0.01	0.154	0.11
Dorsolateral Prefrontal Cortex	−0.08 [−0.22–0.06]	−1.12	0.267			
Dorsolateral Prefrontal Cortex × Sex	0.12 [−0.03–0.28]	1.54	0.125			
Model 3: Stage 1				−01	0.986	0.11
Anterior Prefrontal Cortex	−0.00 [−0.23–0.22]	−0.02	0.986			
Model 3: Stage 2				−0.01	0.676	0.11
Anterior Prefrontal Cortex	−0.03 [−0.42–0.36]	−0.15	0.882			
Anterior Prefrontal Cortex × Sex	0.05 [−0.43–0.52]	0.19	0.850			

The orbitomedial cortex includes the following domains of executive functions: Inhibitory control, follow rules, and risk-taking processing. The dorsolateral prefrontal cortex includes the following domains of executive functions: verbal fluency, mental flexibility, visuospatial planning, sequential planning, reverse sequence, productivity, self-directed visual working memory, verbal working memory-ordering, and visuospatial-sequential working memory. The anterior prefrontal cortex includes the following domains of executive functions: metamemory, comprehension of figurative meaning, and abstract attitude.

*p < 0.01; f^2^ = Cohen’s effect size.

**FIGURE 2:**
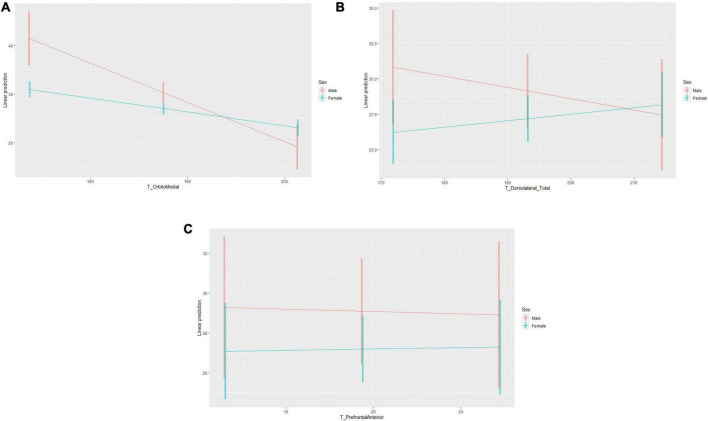
Moderating effect of the sex of the students on the relationship between the executive functions and academic procrastination. **(A)** Moderating effect between the orbitomedial cortex and academic procrastination. **(B)** Moderating effect between the dorsolateral prefrontal cortex and academic procrastination. **(C)** Moderating effect between the anterior prefrontal cortex and academic procrastination.

Regarding the first specific hypothesis, it is observed that the tasks linked to the medial orbital cortex predict a 34% variance of academic procrastination (Δ*R*2 = 0.34; *p* < 0.01). Furthermore, when the dlPFC (*p* = 0.551), aPFC (*p* = 0.998), and age (*p* < 0.05) are included in the model as covariates, the orbitomedial cortex continues to have a significant impact on academic procrastination (*p* < 0.01) and the explained variance of the model remains similar (ΔR2 = 0.36; *p* < 0.01). For the second specific hypothesis, when sex is included as a moderating variable in the model, the degree of explained variance increases significantly (Δ*R*2 = 0.41; *p* < 0.01). It can also be seen that the regression coefficient for the interaction of orbitomedial orbital cortex × sex is significant (β_3_ = 0.53; *p* < 0.01), therefore, the degree of prediction of the medial orbital cortex on academic procrastination depends significantly on the sex of the university students. For men, the estimated effect of the orbitomedial cortex on the degree of academic procrastination is −0.81 (β_1_). For women, the estimated effect of the orbitomedial cortex on the degree of academic procrastination is −0.28 (β_1_ + β_3_). Simple slope analysis shows that the slope of the orbitomedial cortex for males is significantly greater than for females (*p* < 0.01) (see Figure 2). Then, the moderation analysis shows that the effects of tasks linked to the medial orbital cortex on academic procrastination for men and women are significantly different, which is in line with the second specific hypothesis.

Regarding the third specific hypothesis, it can be seen that the tasks linked to the dlPFC fail to predict the degree of academic procrastination (Δ*R*2 = −0.01; *p* = 0.558). It can also be seen that the regression coefficient for the dlPFC × Sex interaction is not significant (β_3_ = 0.12; *p* = 0.154). In addition, the analysis of simple slopes shows that the slope of the dlPFC for men and women is similar (*p* = 0.123). These results provide evidence to reject the third and fourth specific hypotheses.

Regarding the fifth specific hypothesis, it can be seen that the tasks linked to the aPFC fail to predict the degree of academic procrastination (Δ*R*2 = −0.01; *p* = 0.986). It can also be seen that the regression coefficient for the interaction aPFC × Sex is not significant (β_3_ = 0.05; *p* = 0.676). In addition, the analysis of simple slopes shows that the slope of the aPFC for men and women is similar (*p* = 0.849). These results provide evidence to reject the fifth and sixth specific hypotheses.

## Discussion

Regarding the first specific hypothesis, it was shown (step 1) that the tasks linked to the orbitomedial cortex significantly predicts the degree of academic procrastination (Δ*R*^2^ = 0.34; *p* < 0.01). To understand this result, it is essential to point out that the orbitomedial cortex refers to the mPFC and the OFC (Flores Lázaro et al., 2012). The mPFC plays a fundamental role in the processes of (a) regulation and attentional effort (Hauser et al., 2014), (b) decision making between two potentially pleasant outcomes (Saunders et al., 2017), and (c) regulation of motivational states (Fuster, 2002). The OFC also has important processes involved in (a) processing and regulation of affective states (Dixon et al., 2017), (b) behavior regulation (Jonker et al., 2015), (c) change detection (Rolls, 2004), (d) decision-making based on risk-benefit estimation (Zald and Andreotti, 2010), and (e) short- and long-term reward valuation (Peters and D’Esposito, 2016).

Then the processes involved in the mPFC and the OFC can explain the behavior of voluntarily delaying a necessary or important academic activity, despite expecting possible negative consequences that outweigh the positive consequences of the delay. Also, these processes can explain why a failure in inter-temporal choice occurs in procrastination, that is, the tendency to prefer smaller rewards received in the short term to larger rewards received in the long term (Peters and D’Esposito, 2016). In addition, this first result could explain why several previous studies have found that procrastination is related to a failure in self-control (Rebetez et al., 2018; Zhao et al., 2019), in emotional regulation (Eckert et al., 2016; Ljubin-Golub et al., 2019), in the regulation of motivation (Grunschel et al., 2016; Ljubin-Golub et al., 2019) and time management (Wolters et al., 2017).

Regarding the second specific hypothesis, a second analysis (step 2) showed that the tasks linked to the degree of prediction of the orbitomedial cortex on academic procrastination is significantly modulated by the sex of the university students (β_3_ = 0.53; *p* < 0.01). The impact of the tasks linked to the orbitomedial cortex on academic procrastination in males (−0.81) is significantly greater than in females (−0.28). This difference in impact could be related to the fact that neurological structures such as the mPFC and the amygdala, strongly involved in emotional processing and decision making, follow different patterns of functional lateralization in men and women (Reber and Tranel, 2017). In women, decision-making and emotional processing are linked to the left side of the mPFC, while in men, it is linked to the right side of the mPFC (Reber and Tranel, 2017). It could also be related to sex differences in the volume of OFC and mPFC (Gur et al., 2002; Wood et al., 2008). Thus, women show a greater volume of mPFC and right OFC (Welborn et al., 2009). In addition, these structural differences in men and women explain the differences in the use of two emotional regulation strategies: reappraisal and suppression (Welborn et al., 2009). These functional and structural differences could also explain why male procrastinators have higher levels of impulsivity (Strüber et al., 2008), lower levels of self-regulation (Higgins and Tewksbury, 2006) and greater problems planning, monitoring, and evaluating tasks academic (Limone et al., 2020). Unlike women who procrastinate, who have greater problems regulating cognitive and meta-cognitive processes (Limone et al., 2020).

Regarding the third specific hypothesis, it was first evidenced (step 1) that the tasks linked to the dlPFC fails to predict the degree of academic procrastination (Δ*R*2 = −0.01; *p* = 0.558). In addition, for the fourth specific hypothesis, a second analysis (step 2) showed that gender does not play a moderating role in the relationship between both variables (β_3_ = 0.12; *p* = 0.154). To understand these results, it is important to distinguish between hot and cold executive functions. Hot executive functions involve emotion processing and regulation, motivation, reward processing (immediate versus long-term reward), and decision-making based on the subjective value of the reward. While cold executive functions are involved in purely cognitive information processing (Ward, 2020). Several cognitive processes are linked to academic procrastination, such as cognitive flexibility, planning, goal setting, metacognitive skills, and cognitive flexibility (Tan et al., 2008; Rabin et al., 2011; Ziegler and Opdenakker, 2018; Sutcliffe et al., 2019). However, in the present study, other cognitive processes were evaluated, such as verbal fluency, productivity, visuospatial planning, sequential planning, reverse sequencing, and working memory (visual, verbal, and visuospatial). In this sense, the study shows evidence that these domains do not predict academic procrastination. It is important to mention that these domains are linked to the dlPFC (Lamar and Resnick, 2004; Tsujimoto et al., 2004; Ross et al., 2007; Gläscher et al., 2019; Niki et al., 2019; Panikratova et al., 2020; Barahimi et al., 2021), one of the cortical regions associated with cold executive functions. In contrast, the OFC is mainly associated with hot executive functions (Salehinejad et al., 2021). This would explain why the tasks linked to the dlPFC fail to explain academic procrastination, but the tasks linked to the OFC do.

Regarding the fifth specific hypothesis, it was first evidenced (step 1) that the tasks linked to the aPFC fails to predict the degree of academic procrastination (Δ*R*2 = −0.01; *p* = 0.986). In addition, for the sixth specific hypothesis, a second analysis (step 2) showed that gender does not play a moderating role between both variables (β_3_ = 0.05; *p* = 0.676). These results could be because aPFC is mainly related to high-level cognitive functions, such as meta-memory, figurative meaning comprehension, and abstract attitude (Ramnani and Owen, 2004; Flores Lázaro et al., 2008), which are purely cognitive functions. In contrast, academic procrastination is not a problem of cognitive processing but rather an eminently affective, motivational, and processing problem of perceived rewards (Damme et al., 2019).

Regarding the study’s limitations, firstly, a non-probabilistic sampling was used, which limits the generalization of the results. It is recommended that future studies use representative samples to generalize the results. Secondly, the sample size was modest, although sufficient to test the regression models. It is essential to point out that the BAFE 2 allows for an objective evaluation of executive functions, for which an individual evaluation and a minimum of two evaluation sessions are required. This evaluation characteristic could justify the sample size reached in the present study, which was similar to that reported in other studies where the BAFE-2 was used (Rincón-Campos et al., 2019; Muchiut et al., 2021; San-Juan et al., 2022). Third, there was an unequal distribution of genders in the sample, where women were the majority group. Therefore, it is necessary to carry out more studies with balanced samples of men and women and larger and more representative ones to see if the present results can be replicated. Fourth, in the study of the variables, Magnetic Resonance Imaging (MRI) was not included. Therefore, it is recommended that future studies include this type of evaluation to understand the results better. Fifth, covariates such as year of study, among others, were not included in the study. It is recommended that future studies include these variables to understand the results better. Sixth, OFC and mPFC were measured with the same score and under the term orbitomedial cortex. This procedure is directed by the instrument used. Therefore, it is suggested that future studies use instruments that use separate scores for the OFC and the mPFC for a better understanding of the results. Despite these limitations, the study findings are important and promising as it is the first study to assess the moderating role of gender in the relationship between executive functions and academic procrastination using a neuropsychological battery, which allows a more objective evaluation of executive functions, unlike a self-report test.

Based on the above, it is concluded that only the tasks linked to the medial orbital cortex significantly predicts the degree of academic procrastination. In addition, the degree of prediction of the tasks linked to the medial orbital cortex on academic procrastination is significantly moderated by the sex of the university students.

## Data availability statement

The raw data supporting the conclusions of this article will be made available by the authors, without undue reservation.

## Ethics statement

The studies involving human participants were reviewed and approved by Institutional Research Ethics Committee (CIEI) of the Universidad Peruana Cayetano Heredia (204085). The patients/participants provided their written informed consent to participate in this study.

## Author contributions

LV provided initial conception, organization, and main writing of the text, involved in data collection, analyzed the data, and prepared all figures and tables.
